# Supplementary material for: Pulsed low-energy stimulation initiates electric turbulence in cardiac tissue
Source: PLoS Comput Biol. 2021 Oct 8;17(10):e1009476. doi: 10.1371/journal.pcbi.1009476 (PMC8528298; doi:10.1371/journal.pcbi.1009476)
Supplement: Attachment — Submitted filename: rebuttal.pdf [file pcbi.1009476.s005.pdf]

To,

The Editor,

PloS Computational Biology,

Dear Members of the editorial board,

Thank you very much for considering our manuscript “Pulsed low-energy stimulation initiates electric turbulence in cardiac tissue" for publication in PloS Computational Biology.

We have read the comments of the reviewer and have attended to his/her suggestions/questions, as described in detail, below.

Yours sincerely,

Dr. Rupamanjari Majumder,

Dr. Sayedeh Hussaini,

Dr. Vladimir Zykov,

Prof. Stefan Luther, &

Prof. Eberhard Bodenschatz.

****

Response to reviewers:

Reviewer #1: In this study, Majumder et al carried out computer simulations of atrial tissue models to investigate the effects of optogenetically induced subthreshold current stimuli on spiral wave dynamics. They showed that with a constant subthreshold current, the spiral tip meandered with a larger and larger core region as the current strength increases, and no breakup was observed. But when the current with a certain strength was switched on and off, wavebreak occurred. They carried out additional simulations to investigate the mechanism of wavebreak induced by the on-and-off of the subthreshold current stimuli.

Response: Thank you very much for this overall assessment of our work. We would like to add here, that not only have we shown the occurrence of the phenomenon in the human atrial model, but also in the mouse ventricular model. So the results hold true for at least two models of different species.

Reviewer comment 1: The study provides new insights on the role of optogenetics in cardiac conduction dynamics, however, the mechanism of wavebreak was not very clearly demonstrated or explained (see comments below).

Response: We are very sorry, that our description is unclear for the referee and hope, that after some corrections listed below, it becomes more clear.

Reviewer comment 2: The authors were trying to explain the mechanism of wavebreaks by APD (and APD restitution) and CV (and CV restitution) as for spiral wave breakup investigated in many previous studies.

Response: When we first observed wave breaking in our system, our obvious approach was to check whether the APD and CV restitution curves were steep in the parameter regime used, because there is extensive literature linking the breaking of a spiral wave to the steep slope of the APD and CV restitution curves (see Ref. [21] of our present manuscript and the references therein).

Comment 2 (contd.): Although they mentioned the change of excitability during the switch-on and switch-off of the light, I do not think that they explained correctly the "true" mechanism or causes of wavebreak.

Response: Perhaps we have not made ourselves as clear in the paper as we had hoped to have been. Thus, we break down the process of wavebreak formation to five simple steps.

1. Application of light to a single cell (see Fig.4 of our manuscript) prolongs the action potential duration (APD) [1]. The degree of prolongation depends on the phase of the action potential in which the light is applied.

2. When several such cells are connected along a cable and illuminated in the absence of existing electrical activity, all cell membranes respond to the illumination synchronously, and exhibit the exact same response. However, if a signal is propagating through the cable during illumination (see Fig. 5 of our manuscript), then different cells are in different phases of the action potential, when light is applied to them. Thus the light-induced prolongation of APD is heterogeneous among cells constituting the cable.

3. Application of light to a cell cable increases the conduction velocity (CV) of a propagating wave front, but decreases the CV of the corresponding wave back. This causes the wavelength to increase at first. But because of the heterogeneity induced in step 2, a subsequent reshaping of the wave profile occurs (in 1D, this looks like a triangulation of the pulse, followed by a tail of elevated potential).

4. Now, when the light is turned off, the wavefront tries to slow down, the waveback tries to speed up (both attempt to return to their normal velocities). The rate at which the wavefront can slow down is the same as the rate at which the waveback can speed up. But the actual time taken by different cells to repolarize back to their resting values is determined by the state of depolarization of its membrane, within the reshaped wave profile. The tail of elevated potential repolarizes

1 For definition of APD, see our response to next comment.

immediately, and a re-excitable region appears right behind the abrupt triangular pulse (which takes longer to repolarize).

5. In 2D, this re-excitable region provides the base for surrounding excitation to reenter. Thus, a break is formed.

Comment 2 (contd.): One major concern is the definition of APD in Fig.3, which is somewhat ambiguous, even incorrect. First, although APD90 was used, I was not able to find a clear definition of APD in the manuscript. Second, if one uses APD80, APD70, …, then the APD responses to the optogenetic current will be drastically different from what is shown in Fig. 3F. Therefore, which APD is a proper one to use to explain the observed wave dynamics is a problem.

Response: By action potential duration (APD_X) we refer to the amount of time during an action potential, when the membrane voltage is above a threshold value (V_{thresh}). V_{thresh} is calculated as follows:

$$V_{thresh} = V_{max} - \frac{X}{100}(V_{max} - V_{min})$$

Here, V_{max} and V_{min} represent the maximum and minimum values of the membrane voltage, respectively, as recorded during an action potential. X is the degree of repolarization of the cell membrane, that we are interested in looking at.

To provide a clearer description of the APD we have now changed the following text:

"In particular, the maximum conductance for I_{K1} , i.e., G_{K1} was changed from 0.09 nS/pF in the original model, to 0.117 nS/pF, to yield a single cell action potential duration at 90% repolarization of the membrane potential (APD_{90}), of 284 ms."

to:

"In particular, the maximum conductance for I_{K1} , i.e., G_{K1} was changed from 0.09 nS/pF in the original model, to 0.117 nS/pF. This resulted in an APD_{90} value of 284 ms. Here APD_{90} refers to the amount of time during an action potential, when the membrane voltage is less than 90% repolarised. In general, APD_X is calculated as $T_1 - T_0$, where T_1 and T_0 are the time instants at which the membrane voltage crosses the threshold value V_{th} . V_{th} is calculated according to Eq.

$$V_{thresh} = V_{max} - \frac{X}{100}(V_{max} - V_{min}) \qquad ()$$

V_{max} and V_{min} represent, respectively, the maximum and minimum values of the membrane voltage, as recorded during an action potential, and X represents the degree of repolarization of the cell membrane, that we are interested in looking at."

Reviewer comment 3: I do not think that the mechanism of wavebreak is as complex as explained by the authors. It is not related to APD or APD restitution, but to the change of refractoriness or excitability due to the elevation of the resting potential caused by the

optogenetic current. As shown in Figs.3 D and E, the effect of the current in the diastolic phase is to elevate the resting potential. It is well known that elevation of resting potential first increases CV (this is what occurred in Fig.2B for large DI) and then decreases CV due to the competition between Na channel inactivation and the threshold of excitable, i.e., a high resting potential causes more Na channel inactivation but it is closer to the threshold of Na channel activation. A typical example of this is acute ischemia. The spiral wave behavior in response to ischemia (see Figs.1 and 2 in Xie et al, Am J Physiol. 280, H1167(2001)) is very similar to what are shown in Figs.1 A and B in the present study. Another effect of elevation of the resting potential is the prolongation of the recovery time of the Na channel. In the model, it is described by the time constant of the j-gate. This will change the CV restitution (this is why CV decreases faster as DI decreases at very short DIs in Fig.2B) and prolong the effective refractory period to potentiate conduction block. This effect was demonstrated by Xie et al [Heart Rhythm 6, 1641(2009), Fig.7 and Fig.S4] in a study of fibroblast-myocyte coupling in which the resting potential was elevated due to the fibroblast-myocyte coupling. Conduction block caused by elevation of the resting potential was also studied by Liu et al (Heart Rhythm 12, 2115 (2015)) on the role of subthreshold DAD on conduction block.

Response: We would like to point out here that we completely agree with the reviewer. This is also what we had written in the paper. We never claimed the mechanism to be related to APD restitution. As we have summed up in our response to comment 1, the mechanism is related to the elevation (and subsequent repolarization) of the membrane potential, which is caused by heterogeneous response of the excited cells, upon illumination. We have now included some of the references suggested by the reviewer and the following text in the Discussion:

"Here, we attribute the onset of the mechanism of wavebreak initiation to the elevation in the resting membrane potential upon illumination. Elevation of the resting potential results in the prolongation of the recovery time of the Na channel. In the model, it is described by the time constant of the j-gate. This influences the CV restitution at short diastolic intervals and prolongs the effective refractory period to potentiate conduction block [75,76]. Application of light to the domain containing the spiral wave causes the wavefront to speed up, but the waveback simultaneously slows down. This leads to wavefront-waveback interactions which further contribute to the formation of conduction blocks. Our studies show that these conduction blocks appear soon after the light is turned on. Subsequently, existing electrical activity in the doamin to propagate around the blocked regions, resulting in the development of wavebreaks. However, these wavebreaks do not lead to stable reentry. Spiral waves formed around these wavebreaks rotate with much longer periods and larger cores, as compared to spirals formed in the absence of illumination. Thus such spirals tend to meander their way out of the domain within tens of milliseconds. On the contrary, when a wavebreak is formed by switching off the light (as demonstrated in Fig. 2F), the electrical activity within the domain freezes temporarily, allowing the excitable tissue around the break to recover. The spatial distribution of repolarized tissue re-organizes and a spiral is formed with shorter period and smaller core. Thus wavebreaks triggered at the start of the 'dark' period lead to sustained reentry."

Included Refs.:

Xie et al [Heart Rhythm 6, 1641(2009), Fig.7 and Fig.S4]

Liu et al (Heart Rhythm 12, 2115 (2015))

Comment 3 (contd.): Based on the above studies, I believe that the local conduction block or wavebreak is caused by the elevation of the resting potential in a short time after the onset of the optogenetic stimulus current due to the sudden prolongation of the refractory period.

Response: Precisely. We are happy to know that the reviewer agrees with us. This is also what we concluded from our study and reported in our paper.

Reviewer comment 4: In the manuscript, the authors discuss spiral wave breakup and tried to make some link of the wavebreak in the current study to those in the previous studies. Note that the spiral wave breakup occurs as spatiotemporal instabilities (see a review by Qu et al, Phys Rep 543, 61(2014)), which is not the case in the present study. As shown in Fig.1A by the authors, no breakup occurs before the current becomes suprathreshold. Wavebreaks only occur when the current is periodically switched on and off, indicating that wavebreak is caused by a sudden change of the current/parameters with the mechanism explained above.

Response: We are glad to see that the referee consider the observed mechanism of the wavebreak as a new one. It is a serious reason for a publication. Indeed, in our case, the wavebreaks were observed when the system was perturbed, as we have clearly claimed throughout the manuscript. In fact, contrary to what the reviewer concludes from our results, we did see wavebreaks at subthreshold intensities when the light was suddenly applied to a spiral. However, we did not give importance to those findings because we felt that it could be a result of a combination of many factors: system size, parameter instability, transient response etc. To obtain the behaviour that we reported in Fig.1A, we first illuminated the quiescent domain for about 1s, allowing the parameters of the system to stabilize (and to get rid of the initial transients). Then, we initiated a spiral wave in the illuminated domain to see how it evolved in space and time. One could say that high subthreshold perturbations lead to the emergence of dynamical instabilities. However, these instabilities are different from EAD-type instabilities that were reported by Qu et al. Phys Rep 543, 61(2014)).

To clarify this in the manuscript, we have now included the following text in the Conclusion section:

“In this work, the applied subthreshold perturbations of membrane voltage lead to the onset of dynamic instabilities. These instabilities differ from the classical "Early After Depolarization" (EAD) type instabilities that are known to contribute to spatiotemporal chaos in cardiac tissue [77]. Unlike classical spatiotemporal instabilities, which either exist within the system or arise naturally, the instabilities reported in this study are externally induced and occur only in response to discrete

perturbations. Consequently, the system exhibits a stable state characterised by many spiral waves. However, an electrical signal recorded from any part of the domain does not show a broadband frequency spectrum characteristic of classical electrical turbulence.”

Reviewer comment 5: The last sentence in the abstract “This finding changes the paradigm of cardiac arrhythmia research …” is overly stated. It provides some useful insights on optogenetics but not shifts the paradigm of arrhythmia research.

Response: We have now revised this sentence as follows:

“This finding is an important contribution to cardiac arrhythmia research as it identifies scenarios in which low-energy perturbations to cardiac rhythm can be potentially life-threatening.”

We would like to emphasise here that the finding is of great importance for arrhythmia research. In this work, we have demonstrated a mechanism which underlies the triggering of wave breaks during attempted defibrillation. This finding can contribute to the understanding of wave dynamics in cardiac tissue under the influence of small electrical perturbations and can be used to improve defibrillation strategies, which in turn will contribute to advances in arrhythmia management and therapy.

Reviewer comment 6: The sentence in the abstract “This mechanism involves ‘conditioning’ or reshaping the wave profile from front to back, such that, removal of the external light source causes rapid recovery of cells at the waveback, leading to the emergence of vulnerable windows for sustained re-entry in spatially extended systems.” seems to imply that conduction block occurs at the moment of removal of the external light. However, by watching the movie, I saw that conduction block always occurred at the onset of the external light (the moment when the blue circle was on). During the on-phase or the off-phase, the spiral waves are stable (no breakup).

Response: We appreciate the reviewer’s comment. We have revised this line as follows:

“This mechanism involves the creation of conduction blocks through a combination of wavefront-waveback interaction, reshaping of the wave profile and heterogeneous recovery from the excitation of the spatially extended medium, leading to the creation of re-excitable windows for sustained re-entry.”

As we explained in detail in our response to comment 1, just turning on the light causes the wavefront to accelerate and the waveback to slow down. Thus, in the presence of stable spiral wave activity in the domain (where the arms of the spiral are densely packed), high subthreshold illumination can cause conduction blockage due to interactions between the wavefront and waveback in successive turns of the same spiral. Then, as the system evolves, reentry can occur around the break if excitable medium is available.

Reviewer comment 7: Fig.1E should be an expanded figure (as Fig.2) independent from Fig.1. I would suggest to plot voltage snapshots at different time points before, during, and after the switch on and off of the light. It would be also useful to show the period of the spiral wave (from one or two locations) versus time combined with light on and off. I understand that there is an online movie to show the wave dynamics, since the whole paper is about Fig.1E, it is important to show a clear picture or dynamics for the readers. It is not clear to me where and when the wavebreaks occur by reading text only, and it took me sometime by watching the movie to find out that the wavebreaks only occur right after the onset of the light (I hope that I'm correct on this).

Response: We have now included a new figure (Fig.2) in our manuscript, as per the reviewer's suggestion. We have also added the following text in the Results section, in connection with the figure.

"These breaks occur during the illuminated phase or at the beginning of the `dark' phase of the applied stimulus, i.e., when the light was turned off in a pacing cycle. Application of discrete high sub-threshold perturbations to membrane voltage led to the spontaneous emergence of conduction blocks within the domain (see bold white lines indicated by green arrows in Fig. 1E and Fig. 2B,C), soon after light is switched on. These blocks promote wave break initiation. For the full sequence of events leading to the incidence of wavebreaks, see Fig. 2 and Video S1. Voltage time series data from five representative points (128,128), (128,384), (384,384), (384,128) and (256,256) within the 512 x 512 domain are recorded for 5s of simulation. The data shows a large increase in APD and a substantial drop in the period of the electrical activity, during illumination."

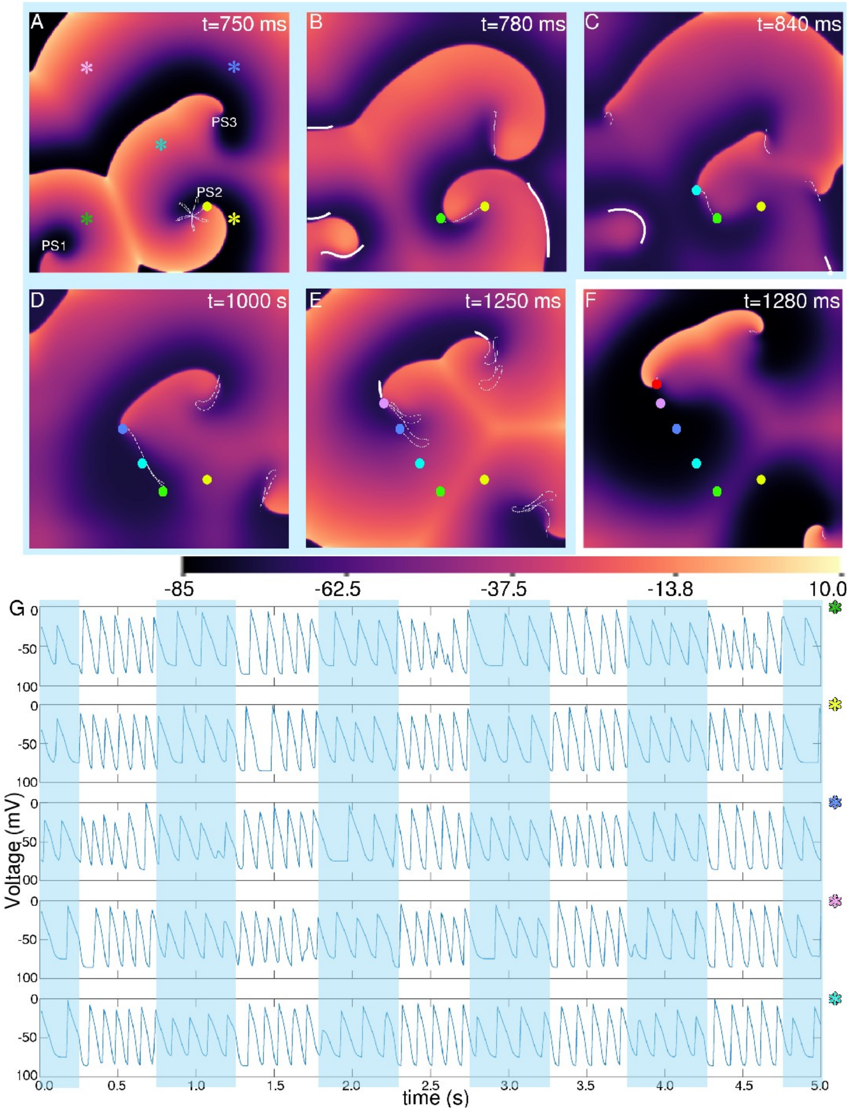

Figure caption:

Demonstration of spiral breakup with pulsed, uniform, global subthreshold illumination, for a light pulse applied from 750 ms, up to 1250 ms. A-F. Spatial distribution of membrane voltage at different time points during (A-E, blue shaded) and after (F, non-shaded) one illumination cycle. The dashed white lines indicate the trajectories of the phase singularities (PS1, PS2 and PS3, shown in A), as they evolve since the preceding time point. Here, we highlight the spatiotemporal evolution of PS2. The hypocycloidal pattern of evolution of PS2 represents the dynamics of PS2 prior to the application of light. The filled yellow, green, cyan, blue, violet and red circles in A-F indicate the positions of PS2 at 750 ms, 780 ms, 840 ms, 1000 s, 1260 s and 1280 s, respectively. The bold white lines mark the zones of conduction block at these particular time points. Absence of a dashed white line in F, together with a sharp increase in the repolarized area, relative to E, show that after removal of light in E the activity remains frozen for a brief period of time (here, 30 ms). This allows the excitable tissue to recover. G. Voltage time series recorded from five representative points within the domain, as marked on A with the pink, green, yellow, blue and cyan asterisk symbols.

Reviewer comment 8: It seems there is a Conclusion section, but the content is missing.

Response: We thank the reviewer for bringing this to our notice. We had somehow overlooked this before. The Conclusion section has now been included in the revised manuscript, as follows:

“In addition to the general notion that suprathreshold perturbations have a profound effect on electrical wave dynamics in cardiac tissue, we demonstrate a situation in which small-amplitude electrical perturbations can lead to wave break initiation and reentry in tissue that otherwise does not exhibit any of the standard markers of excitable medium vulnerability. In this work, the applied subthreshold perturbations of membrane voltage lead to the onset of dynamic instabilities. These instabilities differ from the classical "Early After Depolarization" (EAD) type instabilities that are known to contribute to spatiotemporal chaos in cardiac tissue []. Unlike classical spatiotemporal instabilities, which either exist within the system or arise naturally, the instabilities reported in this study are externally induced and occur only in response to discrete perturbations. Consequently, the system exhibits a stable state characterised by many spiral waves. However, an electrical signal recorded from any part of the domain does not show a broadband frequency spectrum characteristic of classical electrical turbulence. The discovered mechanism has the potential to inhibit low-amplitude defibrillation using pulsed global (electrical or optical) stimulation. In the context of optogenetics, this observation is of crucial value as it provides useful insights into the translational prospects of optogenetic defibrillation in the human atria. The key to success lies in understanding and exploiting the mechanisms of creation and annihilation of spiral waves. Thus, our study makes an important contribution to the advancement of the field of cardiac optogenetics and arrhythmias, in general.”

Have the authors made all data and (if applicable) computational code underlying the findings in their manuscript fully available?

The PLOS Data policy requires authors to make all data and code underlying the findings described in their manuscript fully available without restriction, with rare exception (please refer to the Data Availability Statement in the manuscript PDF file). The data and code should be provided as part of the manuscript or its supporting information, or deposited to a public repository. For example, in addition to summary statistics, the data points behind means, medians and variance measures should be available. If there are restrictions on publicly sharing data or code —e.g. participant privacy or use of data from a third party—those must be specified.

Reviewer #1: **No:** not stated in manuscript where the codes are available.

Response: We have now uploaded our code as supplementary information.
